# Characterization of the complete chloroplast genome of *Camellia brevistyla*, an oil-rich and evergreen shrub

**DOI:** 10.1080/23802359.2019.1703607

**Published:** 2020-01-21

**Authors:** Yupeng Wang, Jiyuan Li, Zhengqi Fan, Dongyang Wu, Hengfu Yin, Xinlei Li

**Affiliations:** a College of Information Science and Technology, Nanjing Forestry University, Nanjing, China;; b State Key Laboratory of Tree Genetics and Breeding, Research Institute of Subtropical, Forestry, Chinese Academy of Forestry, Hangzhou, China;; c Key Laboratory of Forest Genetics and Breeding, Research Institute of Subtropical, Forestry, Chinese Academy of Forestry, Hangzhou, China

**Keywords:** *Camellia brevistyla*, chloroplast genome, phylogenetic analysis

## Abstract

*Camellia brevistyla* is an economic species for its seeds with high oil content and ornamental value, which is cultivated widespreadly across southern China. In this study, the complete chloroplast (cp) genome sequence of *C. brevistyla* was assembled and annotated in order to future genetic research. The whole cp genome of *C. brevistyla* is 159,281 bp in size, composed of a small single copy (SSC) region of 15,662 bp and a large single copy (LSC) region of 86,251 bp separated by a pair of inverted repeats (IRs, IRA: 130598: 159281, IRB: 86252: 114935). The overall GC content of *C. brevistyla* cp genome is 37.19%, with the base content A (31.03%), T (31.78%), C (18.94%), and G (18.25%). Phylogenetic analysis of 20 species based on 74 protein-coding genes shows that *C. brevistyla* is evolutionarily closest to *Camellia danzaiensis*.

*Camellia brevistyla*, one of more than 250 species of the genus *Camellia*, grows mainly in thickets or pine forests on hillsides at an elevation of about 600 m and is found commonly in southeast China. *Camellia brevistyla* is listed as an economic tree species for oil production and horticulture (Su et al. 2017). Meanwhile, terpene synthase genes could be isolated from *C. brevistyla*, which was identified as hedycaryol synthase; it was shown to specifically expressed in flowers and has certain biological application value (Hattan et al. 2016). However, there are few studies on the genomic information and genetic diversity of *C. brevistyla*. Here, we report the first complete cp genome of *C. brevistyla* (Genbank accession: MN640791) and discuss the phylogenetic relationship among its populations, which will provide important reference for its future biological research.

The samples of fresh leaves of *C. brevistyla* were collected from Jinhua International Camellia Species Park (Zhejiang, China; Coordinates: 29°7′10.1208″N, 119°35′52.1088″E). Voucher specimen (CD_01) was deposited in State Key Laboratory of Tree Genetics and Breeding, Research Institute of Subtropical Forestry, Chinese Academy of Forestry. *C. brevistyla* was sequenced using Illumina Hiseq 2500 sequencing systems (Illumina, USA) at Genesky Biotechnologies (Shanghai, China). The 24,696,066 raw reads were under quality control by Trimmomatic (Bolger et al. 2014). The strategy for assembly and annotation the cp genome was adapted from Wang et al. (2018). Finally, the cp genome map of *C. brevistyla* was generated by OGDRAW version 1.3.1 (Greiner et al. 2019).

The complete cp genome sequence of *C. brevistyla* is 159,281 bp with the typical quadripartite structure (Wang et al. 2016), including a SSC region of 15,662 bp, a LSC region of 86,251 bp and a pair of IRs region of 28,684 bp. The total GC content of sequence was 37.19%, and the GC content of IR regions was higher than that of SSC region (30.70%) and SSC region (35.33%). The cp genome of *C. brevistyla* has 131 functional genes, which are composed of 83 protein-coding genes and 48 RNA genes (44 tRNA genes and 4 rRNA genes). The longest genes of protein-coding genes, tRNAs and rRNAs is ycf2_2 with 6867 bp, rrn23 with 2809 bp and tRNA-Ile3 with 239 bp, respectively.

A Minimum-Evolution tree based on 74 protein-coding genes that were screened by the Perl script (Wang et al. 2019) was reconstructed by MEGA7 (Kumar et al. 2016) to study the phylogenetic relationship of 20 *Camellia* species. The evolutionary distances were computed using the JTT matrix-based method (Jones et al. 1992) and there were a total of 22,648 positions in the final dataset. As illustrated in Figure 1, the cp genome of *C. brevistyla* is closest to *Camellia danzaiensis* (NC_022460.1) and also clustered closely to *Camellia pitardii* (NC_022462.1).

**Figure 1.**
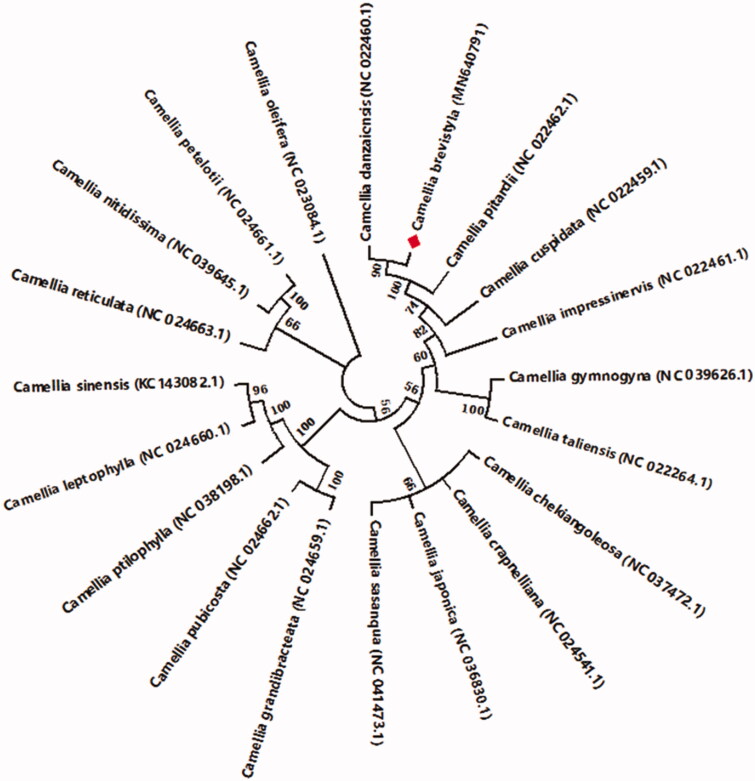
The Minimum-Evolution (ME) tree of 20 *Camellia* cp genomes based on 74 protein-coding genes were conducted in MEGA7. The bootstrap values from 1000 replicates are listed for each node.
